# Patient reported outcomes: looking beyond the label claim

**DOI:** 10.1186/1477-7525-8-89

**Published:** 2010-08-20

**Authors:** Lynda C Doward, Ari Gnanasakthy, Mary G Baker

**Affiliations:** 1 Galen Research Ltd, Enterprise house, Manchester Science Park, Lloyd Street North, Manchester, M15 6SE, UK; 2 Global Health Economics and Outcomes Research, Novartis Pharmaceuticals, New Jersey, USA; 3 European Federation of Neurological Associations, 69 East King Street, Helensburgh, G84 7RE, UK; 4 European Brain Council, Fondation Universitaire, 11 Rue D'Egmont, B-1000 Bruxelles, Belgium

## Abstract

The use of patient reported outcome scales in clinical trials conducted by the pharmaceutical industry has become more widespread in recent years. The use of such outcomes is particularly common for products developed to treat chronic, disabling conditions where the intention is not to cure but to ameliorate symptoms, facilitate functioning or, ultimately, to improve quality of life. In such cases, patient reported evidence is increasingly viewed as an essential complement to traditional clinical evidence for establishing a product's competitive advantage in the marketplace. In a commercial setting, the value of patient reported outcomes is viewed largely in terms of their potential for securing a labelling claim in the USA or inclusion in the summary of product characteristics in Europe. Although, the publication of the recent US Food and Drug Administration guidance makes it difficult for companies to make claims in the USA beyond symptom improvements, the value of these outcomes goes beyond satisfying requirements for a label claim. The European regulatory authorities, payers both in the US and Europe, clinicians and patients all play a part in determining both the availability and the pricing of medicinal products and all have an interest in patient-reported data that go beyond just symptoms. The purpose of the current paper is to highlight the potential added value of patient reported outcome data currently collected and held by the industry for these groups.

## Introduction

In recent years, the pharmaceutical market has become characterized by more knowledgeable customers, growing cost pressure from private and public third party payers and increasing need for product differentiation in a highly competitive market. To ensure product success companies must generate value propositions that go beyond traditional safety and clinical efficacy messages. One route to achieving this is by generating evidence on the patient's perspective of treatment. Such evidence is commonly generated by using patient reported outcome (PRO) measures to assess patient views on product efficacy. PRO is an umbrella term used to describe outcomes collected directly from the patient without interpretation by clinicians or others[1-3]. PRO data are collected via standardised questionnaires designed to measure an explicit concept (construct) such as symptoms, functioning (activity limitations), health status/health related quality of life (HRQL) or quality of life (QoL).

Industry-sponsored PRO use centres predominantly around inclusion in marketing studies, patient registries and clinical trials. Although PRO endpoints are still used in a minority of clinical trials their use has grown in recent years, particularly in randomised Phase III trials. An analysis of clinical trials registered with ClinicalTrials.gov shows that approximately 12% of the interventional trials registered by the pharma industry and over 15% of non-industry sponsored protocols now incorporate some form of PRO assessment[4]. Although for certain therapeutic areas (most notably, psychiatric disorders) PROs may be included in clinical trials as primary efficacy indicators, commercial use of PRO outcomes focuses predominantly on their employment as secondary endpoints designed to provide 'added value' data to support key biomedical endpoints. Such 'value' is viewed largely in terms of their potential for securing a labelling claim in the USA or inclusion in the summary of product characteristics (SmPC) in Europe and in providing supporting arguments for reimbursement. Indeed, since the publication of the US Food and Drug Administration's (FDA) Guidance on use of PROs to support potential claims in product labelling, discussion on PROs and label claims has received considerable attention both within the literature and at industry or professional society meetings[5-7]. Consequently PROs are routinely included in clinical trials with these objectives in mind and the data used solely for these purposes. However, regulators and payers are only two of the key stakeholders with an interest in the drug licensing and reimbursement process. Both clinicians and patients now play a key role in influencing the availability and use of pharmaceutical products. Indeed, it is the interaction between these interested bodies that can help or hinder a drug's progress to market and ultimately, its success as a product. The focus for the current study is to look at whether the pharmaceutical industry maximises the potential for generating PRO-based added value messages both in terms of the quality of the data collected and the relevance of those data for key stakeholders. Ultimately, this study aims to highlight the value in considering the use of PRO data beyond acquiring a label claim.

## Discussion

### Establishing an effective PRO strategy

The formulation of an overall PRO strategy for a development compound is a critical but often overlooked step in the development of a high-quality value product proposition package. The added value of PROs to the product development strategy rests on the use of high quality scales that address constructs of interest to the target audience, with appropriate measurement, data capture and reporting strategies. However, an effective strategy requires a clear understanding of PROs to enable a judgment of what they actually measure (and hence, which stakeholders will be interested in the resultant data) and how to assess their quality. Furthermore, it requires company commitment to planning, often to thinking 'outside the box' rather than the replication of former approaches. Unfortunately, criteria for PRO scale selection are occasionally restricted to issues of availability or familiarity rather than considerations of instrument relevance or quality. The use of inappropriate instruments and the lack of explanation for the choice of instruments in clinical trials has been a constant complaint by many authors[8-11]. In some disease areas, such as plastic reconstructive surgery and liver transplantation inappropriate PROs are included in studies since there are no gold standard instruments available [12,13]. Occasionally, certain PRO instruments are selected ahead of more appropriate scales because they are considered to be a 'standard' in that disease area (for example, the Dermatology Life Quality Index in psoriasis)[14]. Consequently, trials do not always use the most appropriate or highest quality instruments[15]. Fundamentally, an effective strategy requires the allocation of sufficient time and resources. Aggressive company timelines can affect the feasibility of the best-designed strategies and it is not uncommon for companies to approach PRO consultants with insufficient time for advice to be implemented. This is particularly noted where there is a need to develop a new instrument or provide new language versions of an existing PRO scale.

### What do PROs measure?

**Figure 1:**
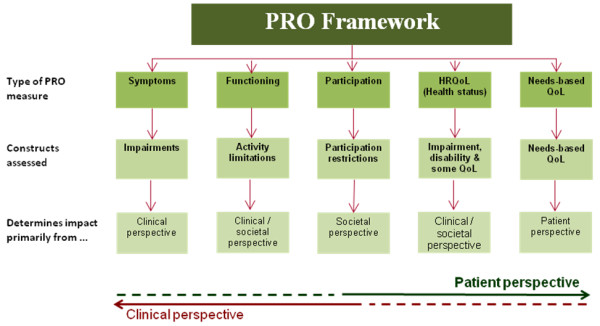
**Conceptualization of PRO constructs**.

A well-designed PRO questionnaire should inform on an explicit PRO concept; that is, the construct addressed by the measure should be clearly stated by the instrument authors. PROs commonly used as endpoints in clinical trials and studies include measures of symptoms, functioning (activity limitations), health status/HRQL and QoL (Figure 1). More recently, trials have also incorporated PROs that address patient satisfaction, compliance and treatment preferences. Measures of symptoms address 'impairments'; that is, any loss or abnormality of psychological, physiological or anatomical structure or function[16,17]. Measures assess a deviation from an individual's normal biomedical status, informing on symptoms and on the adverse effects of interventions. Examples include measures of anxiety, pain and cough. Activity limitation PROs address physical, social or psychological functioning; that is, any restriction or lack of ability to perform an activity in the manner or within the range considered normal for a human[16,17]. Examples include assessments of activities of daily living such as dressing, walking or personal care. HRQL has been defined as 'the capacity to perform the usual daily activities for a person's age and major social role'[18]. Thus, deviation from normality results in a reduced HRQL. Its emphasis is on the measurement of a combination of symptoms and functioning and, as such, HRQL relates to health status. Consequently, measures of HRQL are multi-dimensional, yielding a profile of scores. The outcome of QoL is considered to be a substantively different outcome from HRQL[19]. The most widely implemented approach to the measurement of QoL is the needs-based model of QoL. This postulates that individuals are driven or motivated by their needs and that the fulfilment of these provides satisfaction[2]. Consequently, life derives its quality from the ability and capacity of the individual to satisfy certain human needs. For example, the function of walking may lead to the satisfaction of several needs including socialisation, independence and communication. Needs-based QoL measures are unidimensional and thus, yield a single score[2].

### Are all PROs equal?

Determining the appropriate construct for assessment alone does not guarantee a successful PRO evaluation strategy. Selecting the most advantageous instrument also requires consideration of key quality standards. There are currently thousands of PRO instruments available to users. The Patient Reported Outcomes and Quality of Life Instrument database (PROQOLID) currently includes 654 PRO instruments, over 500 of which are condition-specific scales[20,21] while the On-Line Guide to Quality-of-Life Assessment (OLGA) is reported to include thousands of PRO scales[22,23]. However, a PRO instrument is only of value if it is well designed. Instruments should be based on a sound, theoretical model of what they measure. Without this, it is impossible to conclude that the measure has construct validity[24]. Measures should be derived from direct patient-input to ensure their relevance to the study population and possess adequate psychometric and scaling properties[2,25]. Instruments should adequately sample or cover content relevant to the construct assessed (content validity). A well-designed PRO is capable of assessing patients across a broad spectrum of disease severity. Conversely, poorly designed PROs may be incapable of identifying changes in the construct measured associated with treatment for very mild or very severe patients. PROs that are highly relevant to the patient group under study will maximise the quality of the data collected. Irrelevant questionnaire content can alienate respondents, making them feel that their views are not fully appreciated. This can lead to missing data; respondents may fail to answer questionnaires they consider irrelevant and disaffected respondents may take less care completing the questionnaire and miss additional questions in error. Generic scales, by definition, contain some questions that are irrelevant to specific patient groups and miss areas of particular importance. The use of well-designed disease-specific scales ensures that patients are only asked questions that are relevant, meaningful and acceptable to them. PROs used in any study designed to measure change should also have excellent reproducibility. It should be noted that internal consistency as assessed by Cronbach's Alpha does not inform on scale reproducibility but rather provides an indication of the interrelatedness of questionnaire items[26]. Furthermore, any scale (or sub-scale) for which the questions are summed to produce a single score must be unidimensional. That is, it should measure a single underlying concept. Item Response Theory, (predominantly Rasch analysis) is now considered to be the most efficient means of establishing unidimensionality[27-30].

Where instruments are required for multi-country studies, as is the case for most clinical trials, it is essential to ensure that the different language versions have been translated using suitable methods and their psychometric properties established[31,32]. However, a particular challenge to global clinical development programmes is the *relevance *of the content of a PRO scale to the culture and lifestyle of all country centres. While it is usually possible to translate a PRO questionnaire into a new language, it does not follow automatically that the content is relevant or suitable for the target culture. For example, content on sexual behaviour that is considered suitable by Western European respondents may be considered offensive by respondents in Southeast Asia. Cultural relevance should be formally assessed and documented for all translated versions of PRO scales.

The selection of the most appropriate PRO scales for the trial programme may necessitate the development of new language versions of the questionnaire. This is particularly the case for some of the more recently developed condition-specific scales. Given the expense of, and time required for translation and psychometric assessment of additional countries careful consideration should be given to selecting the countries in which a trial will be run. Collecting PRO data from a large number of countries where individual samples may be small is less efficient than selecting larger numbers of participants from fewer countries where validated versions of the outcome measures are already available.

The ability to produce high quality PRO instruments has advanced considerably in recent years[25]. Furthermore, the increased need for researchers to document and standardise their research practices has led to a drive for higher quality scales. It is not enough to select a measure for a study based on previous use in the disease area. Selection should be based on both the relevance of the content and the suitability of scale psychometric and scaling properties in order to ensure that the scale has the ability to measure change. In particular, the continued use of older generic scales such as the Nottingham Health Profile (NHP)[33], the Short Form 36 (SF-36)[34] and the EuroQoL-5D (EQ-5D)[35] as measures to report the patient-perceived effects of treatment is often questionable. In addition to the relevance issues highlighted above, such scales, being older, pre-date the advances in measurement science that have taken place over the past decade. The NHP and the SF-36 in particular, were designed for use in cross-sectional population studies and, as such, lack the psychometric and scaling quality expected of modern instruments designed to measure change. Inviting patients to complete instruments that have limited ability to demonstrate the perceived effects of treatment raises serious ethical questions. The use of poorly designed instruments results in a wasted opportunity to demonstrate PRO outcomes at a time when the industry can ill afford to squander precious financial resources.

### The value of PROs to key stakeholders

The audience with an interest in PRO outcomes has broadened in recent years to include not only patients and their representatives but also regulators, policy makers, health technology assessment (HTA) authorities and physicians. All of these stakeholders play a part in determining both the availability and the pricing of medicinal products and all have an interest in patient's views on the effects of treatment. This is particularly apparent for products launched in the economically advanced nations whose health care systems are predominantly concerned with the treatment of chronic, disabling conditions associated with an ageing population in a climate of restricted financial resources. Where products are designed to improve life quality rather than to cure, the communication of patient-perceived effects can provide a valuable adjunct to measures of clinical efficacy and aid product differentiation. The question remains, what PRO outcomes are of greatest interest to key stakeholders.

#### The regulatory perspective

The key regulatory authorities have expressed interest in seeing PRO data included within product submissions. While the FDA has produced formal Guidance on the use of PROs to support potential claims in product labelling, the European Medicines Agency (EMA) has opted not to issue similarly formal guidance at this time. Instead, EMA has produced a Reflection Paper to provide broad recommendations on the use of PRO measurement in the context of existing guidance documents[36]. Following on from this, EMA has now launched a Biomarker's Qualification programme to provide a formal mechanism for ratifying clinical trial endpoints, including new or existing PROs[37].

Although the FDA's advisory committees have requested PRO data to be collected in clinical trials it appears that they favour symptoms-based PROs for label claim submissions over other potential PRO endpoints. A review of PRO labels for drugs approved in 2007 and 2008 showed that 75% of PRO label claims were granted for signs and symptoms, 13% for activity limitations and 13% for HRQL[38]. Conversely, EMA currently appear to take a more flexible approach. For the same period EMA included signs and symptoms-based PROs in 55% of SmPCs authorised, activity limitations endpoints were included in 14% and HRQL endpoints in 31% of SmPCs[38]. EMA disease-specific guidelines frequently request PRO endpoints ranging from symptoms to QoL data to be included as key secondary end-points. While specific questionnaires are occasionally suggested (for example, the Ankylosing Spondylitis Quality of Life Questionnaire; ASQoL)[39]. EMA appear to be open to the inclusion of any scale providing it has been appropriately developed, has adequate psychometric properties and its use can be justified for the study population. Of the 81 final clinical guidance documents currently available (for the following disease categories/body systems: Alimentary tract and metabolism, Cardiovascular system, Dermatologicals, Genito-urinary system and sex hormones, Anti-infectives for systemic use, Antineoplastic and immunomodulating agents, Musculo-skeletal system, Nervous system, and Respiratory system) from the EMA website, 39 specified guidelines for PRO inclusion as either primary (n = 5), secondary (n = 22) or both (n = 12) trial endpoints[40].

The position of the regulatory bodies, and the FDA in particular, has caused much debate in the pharmaceutical and health outcomes communities. International learned societies have held workshops to debate its impact and journals have hosted special issues devoted to the topic[41]. While this debate has highlighted quality issues for PROs, it appears to have shifted attention, almost exclusively, to the use of PRO data in pursuit of the label claim. However, it would be unfortunate if this debate removed the focus from the true purpose of PRO data collection; that is, to inform on the patient's perspective on the effects of treatment.

#### HTA and reimbursement authorities

Heath Technology Assessments (HTA) are used in many countries to determine the benefits or added value of new technologies for the purpose of reimbursement and pricing decisions and/or the establishment of clinical guidelines[42,43]. As health expenditures soar these bodies are increasingly concerned with assessing value for money; particularly for new and potentially expensive pharmaceutical products. Several countries now have formal agencies with the specific remit of evaluating the relative clinical and economic benefits of drug therapies. In Canada, Australia and many parts of Europe societal or patient perspectives are included as part of the HTA process[44,45]. For example, patients' organizations are involved in all aspects of the consultation process of the German Institute for Quality and Efficiency in Health Care (IQWiG). Similarly, the UK National Institute for Clinical Excellence (NICE) has made a public commitment to include the views of patients, voluntary organisations and the general public in order to produce guidance that reflects their views [46]. Indeed, patient pressure was a key factor in the decision by NICE to approve Herceptin, a treatment for early stage breast cancer, for use by the National Health Service[47].

The HTA's largely recommend both quantity and quality of life measurement parameters as part of their evaluations. For example, NICE has recommend patient scores the QoL in Adult Growth Hormone Deficiency scale (QoL-AGHDA)[48] as one of the three criteria for judging patient suitability for treatment with recombinant human growth hormone[49]. Similarly, scores on the Dermatology Life Quality Index (DLQI) are used to judge suitability for drug treatments for psoriasis and eczema[14,50-52].

Although no formal HTA agency exists as yet in the USA, the recent interest in comparative effectiveness research has prompted US commentators to call for the HTA process to include not only of clinical outcomes, but also "... important measures of effectiveness such as patient-reported outcomes, including health related quality of life, patient satisfaction, activities of daily living, and work productivity as relevant to the various USA stakeholders."[53]. Indeed health payers now commonly seek the input of patient information (either via direct views or PRO data) in making reimbursement decisions. For example, the importance of addressing subjective PRO outcomes was emphasised last year by WellPoint, one of the largest US health benefits companies. WellPoint has issued formulary guidance to give drug companies more detailed advice on submitting information on a drug's cost-effectiveness and its impact on pharmacy and medical budgets, as well as its effectiveness in improving patients' quality of life[54]. Indeed, In search of patient-centric evidence WellPoint carry out its own outcomes studies to make formulary decisions[55].

#### Clinical perspective

Clinical-rating scales, whereby the physician completes a form to rate disease severity or treatment effects, have long been employed in clinical practice. However, there are often wide discrepancies between patient and clinical views of treatment effectiveness[56-58]. Clinicians often report fewer problems than patients, may underestimate the severity of the problems and overestimate treatment improvement[59-62]. For example, discrepancies have been demonstrated between clinical and patient based reports of pain and overall health in rheumatoid arthritis patients, with clinicians consistently rating pain levels as lower and health status as higher than patient ratings[63]. Similarly, for cancer patients general practitioners have reportedly rated pain as up to 40% lower than the patient-based ratings on up to 57% of occasions. Physicians have also been shown to consistently underestimate the QoL of breast cancer patients[64]. As a result, there is a growing awareness of the need to take account of the patient's views in the healthcare evaluation process and the use of PROs by physicians is growing. Indeed, there have been calls to include PROs as part of routine patient assessment in clinical practice; either for screening purposes or to aid management of individual patients[65]. However, the specific PROs of interest to clinicians do not always correspond to those of interest to patients. For example, one of the commonly used measures of activity limitations for Ankylosing Spondylitis (AS), the Leeds Disability Questionnaire[66] enquires about the patient's ability to look for objects on high shelves. However, interviews conducted with AS patients as part of a study to develop a QoL scale, revealed that AS patients organise their lives so that they never have the need to use high shelves. Although the ability to crane one's neck may be an important issue for clinicians to consider, such physical limitations may be of little concern to the patient[67].

Despite these observations, publication of PRO data can demonstrate drug benefits to clinicians. For example, the Pfizer International Metabolic Database (KIMS) collects data on both treated and untreated adults with growth hormone deficiency (GHD) to provide evidence based medicine to clinicians. Since its inception in 1984, KIMS has routinely collected QoL data using the QoL-AGHDA questionnaire[68]. PRO data have also been used to predict survival and fatigue reported by cancer patients has been shown to be a predictor of survival[69,70]. PROs can be used to better understand patients' symptom experience and satisfaction. This will in turn can improve health professionals' symptom appraisal efforts, enabling them to provide better quality of care and encourage compliance.

#### The patient perspective

Patients' involvement in the care they receive is undoubtedly being given greater emphasis. Indeed, there have been calls to embrace patients as partners in the evaluation of healthcare technologies[71,72]. The American College of Physicians has declared that the patient has a right to self determination and the World Health Organisation has stated that patient involvement in their health care is not only desirable but a social, economic and technical necessity[73,74]. Patients want to be involved in the decision making process, especially when alternative treatments exist[75]. Patients have ultimate responsibility for many decisions taken in connection with their health. Specifically, they decide *when *to seek medical advice, *whether *to accept that advice and ultimately whether to comply with prescribed medicines or whether to present a case for an alternative product. Consequently, the patients' voice in relation to outcomes is being taken far more seriously by health payers and policy makers[76].

As discussed above, a well-designed PRO strategy for a development compound should include measures of relevance to patients. It should be noted that patients can (and generally will, if asked) complete any form with which they are presented. This does not suggest that the information collected by the questionnaire is necessarily of interest to or of value to them. Indeed, discrepancies exist between the specific outcomes of interest to patients and clinicians[57,58]. For example, patients with systemic lupus erythematosus base their assessments of their disease activity on its psychological and broader QoL impact, whereas clinicians base their assessment on its physical effects[77,78]. Measures of symptoms, functioning and HRQL can provide valuable information about the level of impairment or disability experienced by the patient to complement physician ratings in these areas. However, they provide a framework for assessing interventions predominantly from a clinical rather than patient perspective. Patients with chronic disease adapt to their condition, often replacing activities that they can no longer perform with others that are equally satisfying. For example, a multiple sclerosis patient with ambulatory problems can maintain a reasonable level of QoL by remaining independent through the use of a walking frame or wheelchair. Function-based measures are unable to cope with such adaptation making it difficult for severely ill or disabled patients to show improvement, even following effective interventions. Indeed, the emphasis placed on physical functioning in HRQL instruments determines that disabled people cannot have a good 'QoL'; a fact that is not borne out by experience. HRQL should not be confused with QoL. Bradley argues that 'clinicians may be misled into thinking that findings based on a HRQL instrument indicate that treatments do not damage QoL when all the data reveal is that treatments do not damage perceived health'[79]. QoL measurement goes beyond the impairments and activity limitations assessed by HRQL instruments[80,81]. To obtain a complete picture of the impact of disease and of the effectiveness of treatment from the patient's perspective, particularly when a product cannot promise to cure or to extend a patient's life, assessment of QoL becomes paramount.

Undoubtedly, pressure from patients and patient advocacy groups is one of the main driving forces behind the increased focus on PROs. Capturing patients' experience, needs and concerns in product labels has become increasingly challenging. The FDA may be unwilling to consider PRO data beyond first order impacts (signs and symptoms). However, it is clear when talking to patients and patient groups that such concerns are often of minor concern to their determination of the impact of disease and the effectiveness of treatments. Patients have very real ideas about what states of physical and emotional well-being (and ultimately QoL) are acceptable and may not always agree with clinicians and regulators on whether treatments are beneficial. This point should not be disregarded lightly. As it becomes increasingly common for pricing models to incorporate patients' views on the value of products, these may be taken into account even where they contradict those of other stakeholders[82].

### Does the industry make full use of its PRO data?

A PRO strategy for a new compound requires companies to consider all potential means of making interested parties aware of relevant information. Strategies for dissemination of key messages will need to evolve to keep pace with developments in emerging methods of communication. The key to making the best use of PRO data is to disseminate those data as widely as possible to all key stakeholders. Despite the increasing use of PRO endpoints in clinical trials, patient registries and marketing studies, much of the data collected remains underutilised and frequently, under or even unreported. Irrespective of whether a successful PRO-based label claim is achieved, PRO data collected in trials should be published in peer-reviewed academic journals. Too often PRO data considered unsuitable for a label claim by regulatory authorities are cast aside by the industry as unworthy of further attention. Certainly, investigators often find it difficult to justify the resources required to prepare a publication or to conduct valuable secondary analyses. However, key stakeholders are interested in PRO-evidence. As clinicians in particular become more leery of traditional sales methods, academic publications become a crucial vehicle for presenting product value messages[82]. However, information contained in such publications has always been accessible only to those professionals lucky enough to be in an institution that subscribes to a particular journal. The availability of emerging technologies has effectively broadened the audience able to access such information. Patients in particular are keen to identify information on those treatment benefits that are of interest to them - and even keener to disseminate useful findings through web-based networking sites.

In addition to providing data on treatment efficacy, secondary analysis can be conducted on PRO data collected in clinical trials to provide disease or drug intelligence. An exploration of the key demographic (age, gender etc.) and clinical factors (duration, severity, diagnostic groupings etc) influencing for example, patient perceived severity of condition, functional impact or QoL can further our understanding of the disease from the patient's perspective[83]. This can provide an exploration of key factors that predict and explain functional and QoL impact, information on mediating factors in disease severity and implications for treatment, especially product targeting. Secondary analysis can provide market intelligence effectively at a reduced cost (as the data collection has been conducted for other purposes) that can be fed into company strategies for targeted drug development and marketing. Again, disseminating such information via peer-reviewed academic journals and supporting dissemination via Internet-based technologies is to be encouraged.

Presenting well thought out PRO-based information, whether this relates to product effectiveness or disease intelligence, demonstrates company commitment to patients and enhances the company's reputation with patient groups and clinicians. The question that the industry should be asking itself is "are we making the best use of the data we collect and hold"?

## Conclusions

The inclusion of PRO data in label claim submissions is likely to remain for some time the key goal of PRO-endpoints use in clinical trials. Nevertheless, there are limitations to the use of such data in this context; not least the preference of the FDA for symptom-based data. Although key stakeholders, including patients, place high premium on PROs the new regulatory guidance places high hurdles for companies to make claims beyond symptom improvements. However, the value of PROs goes beyond satisfying requirements for an FDA label claim. EMA, payers both in the US and Europe, clinicians and patients and their representatives all have an interest in PRO data that go beyond just symptoms. The competitive advantage lies in identifying broader PRO outcomes that are relevant to key stakeholders, identifying the best possible measures to assess these and in finding the most innovative ways of communicating PRO-value messages.

As the industry can no longer rely on traditional pharmaceutical sales models alone companies are increasingly looking to new forms of communication technology to demonstrate the value of products to a wider audience beyond the traditional physician pool. While a QoL label claim may be illusive in the current climate, the publication of an article demonstrating the benefits of a drug treatment based on data from a well developed PRO scale is likely to have a far reaching impact. The publication of data based on such PROs is likely to find its way onto patient-web sites and such information **is **of interest to both patients and patient advocacy groups alike. Furthermore, these are precisely the kind of data that patient advocacy groups feel they need in order to lobby payers and politicians in order to gain access to newer, often more expensive medical products.

## Competing interests

The authors declare that they have no competing interests.

## Authors' contributions

LCD and AG were involved in the design and drafting of the manuscript. MB reviewed and contributed to the production of the manuscript. All authors read and approved the final manuscript.

## Authors' information

Lynda Doward is Director and Principal Researcher at Galen Research. She has over twenty years experience in the health outcomes field, specialising in the development of disease-specific PRO instruments. The research team at Galen are at the cutting edge of innovation in PRO development; advancing the science of measurement and improving PRO quality standards. The team have produced over thirty PRO scales that have been adapted for use in over sixty languages. Ms Doward has published widely in peer reviewed journals. She has lectured throughout the world and provided advice and guidance to pharmaceutical companies, medical personnel and academic researchers on the incorporation of outcome measurement into pharmaceutical product development strategies, clinical trial design and questionnaire development, translation, adaptation and validation.

Ari Gnanasakthy is an Executive Director at Novartis Pharmaceuticals. He has been in the pharmaceutical industry for almost 20 years. Within Novartis he has been in various functions including Biostatistics, Health Economics and Outcomes Research. In his current role in Novartis Ari acts as an internal consultant when brand teams assess the potential of PRO assessments in compounds in development.

Mary Baker, MBE, has worked for 18 years advocating the needs of people living with Parkinson's Disease (PD) and their families and developing methods of good practice. She is also a former President of the European PD Association (EPDA) and a former Chief Executive of the PD Society of the United Kingdom. In addition, Mrs Baker is a former patient editor of the British Medical Journal (BMJ), Chair of the BMJ Patient Advisory Group, member of the ABPI Code of Practice and a member of the Management Board of the European Medicines Agency (EMEA).
